# Economic Evaluation of First-Line Treatments for Metastatic Renal Cell Carcinoma: A Cost-Effectiveness Analysis in A Health Resource–Limited Setting

**DOI:** 10.1371/journal.pone.0032530

**Published:** 2012-03-08

**Authors:** Bin Wu, Baijun Dong, Yuejuan Xu, Qiang Zhang, Jinfang Shen, Huafeng Chen, Wei Xue

**Affiliations:** 1 Clinical Outcomes and Economics Group, Department of pharmacy, Renji Hospital, affiliated with the School of Medicine, Shanghai Jiaotong University, Shanghai, People's Republic of China; 2 Department of Urology, Renji Hospital, affiliated with the School of Medicine, Shanghai Jiaotong University, Shanghai, People's Republic of China; 3 Department of Oncology, the Second Hospital of Nanjing, affiliated to Medical School of South East University, Nanjing, People's Republic of China; 4 Department of Oncology, Shanghai Putuo Hospital, Shanghai University of Traditional Chinese Medicine, Shanghai, People's Republic of China; Deutsches Krebsforschungszentrum, Germany

## Abstract

**Background:**

To estimate, from the perspective of the Chinese healthcare system, the economic outcomes of five different first-line strategies among patients with metastatic renal cell carcinoma (mRCC).

**Methods and Findings:**

A decision-analytic model was developed to simulate the lifetime disease course associated with renal cell carcinoma. The health and economic outcomes of five first-line strategies (interferon-alfa, interleukin-2, interleukin-2 plus interferon-alfa, sunitinib and bevacizumab plus interferon-alfa) were estimated and assessed by indirect comparison. The clinical and utility data were taken from published studies. The cost data were estimated from local charge data and current Chinese practices. Sensitivity analyses were used to explore the impact of uncertainty regarding the results. The impact of the sunitinib patient assistant program (SPAP) was evaluated via scenario analysis. The base-case analysis showed that the sunitinib strategy yielded the maximum health benefits: 2.71 life years and 1.40 quality-adjusted life-years (QALY). The marginal cost-effectiveness (cost per additional QALY) gained via the sunitinib strategy compared with the conventional strategy was $220,384 (without SPAP, interleukin-2 plus interferon-alfa and bevacizumab plus interferon-alfa were dominated) and $16,993 (with SPAP, interferon-alfa, interleukin-2 plus interferon-alfa and bevacizumab plus interferon-alfa were dominated). In general, the results were sensitive to the hazard ratio of progression-free survival. The probabilistic sensitivity analysis demonstrated that the sunitinib strategy with SPAP was the most cost-effective approach when the willingness-to-pay threshold was over $16,000.

**Conclusions:**

Our analysis suggests that traditional cytokine therapy is the cost-effective option in the Chinese healthcare setting. In some relatively developed regions, sunitinib with SPAP may be a favorable cost-effective alternative for mRCC.

## Introduction

Renal cell carcinoma (RCC), the most common type of kidney cancer, accounts for about 3% of all human malignancies. It is estimated that nearly 30% of the patients with RCC have distant metastatic disease at presentation and that half of those with localised disease subsequently develop metastases during the course of their disease [1], [2]. The median overall survival for metastatic RCC patients is 10 months, and the 5-year survival rate is 5–15%, even when all visible disease is cleared by metastasectomy and nephrectomy [3], [4]. RCC is insensitive to traditional cytotoxic agents and radiation therapies. At present, the most widely used regimens for metastatic RCC (mRCC) are cytokine therapies, including interferon-alfa and interleukin-2, which in previous studies showed response rates of only 10–20% and resulted in debilitating adverse effects [5], [6], [7]. Studies of cytokine therapies with or without chemotherapy have shown short-term partial response rates of up to 20–35% [8], [9]. New treatments are needed to develop strategies for controlling metastatic disease and improving quality of life.

Recently, targeted pharmacological therapies (e.g., sunitinib, sorafenib, bevacizumab, pazopanib, axitinib and temsirolimus) have been developed for RCC treatment [10]. Among them, sunitinib and bevacizumab (combined with interferon-alfa) have been accepted as first-line therapy options for patients with mRCC. Sunitinib is an orally administered multi-target inhibitor of vascular endothelial growth factor (VEGF) and platelet-derived growth factor (PDGF); bevacizumab is a humanised monoclonal antibody that selectively inhibits VEGF-A, which is involved in cancer angiogenesis. These targeted therapies can improve progression-free survival (PFS) and overall survival (OS) [11], [12], [13], [14]. However, their substantial cost restricts their widespread use, especially in health resource–limited regions. An evaluation of the cost-effectiveness of these new therapies is important for improving resource allocation efficiency. A few economic studies of these new therapies have been reported [15], [16], [17], [18]. However, there have been few cost-effectiveness analyses conducted in resource-limited settings, where the front-line therapy for mRCC still involves traditional chemotherapy and cytokine therapy.

In the current study, we evaluated the long-term economic outcomes of five first-line mRCC regimens based on clinical practice and recommendations: [19] sunitinib, bevacizumab plus interferon-alfa, interferon-alfa, interleukin-2, and interleukin-2 plus interferon-alfa. Because the follow-up times of most clinical trials do not focus on the lifetime course of the disease, head-to-head comparisons among several different therapies are rarely reported. Thus, mathematical modelling techniques must be used to supply decision making information. A perspective of Chinese healthcare system was adopted to assist in determining the direct economic value of the five different first-line therapies, given the willingness-to-pay threshold of Chinese and Shanghai residents that is associated with the per capita GDP per quality-adjusted life-year (QALY) gained. The analysis excluded indirect societal costs (i.e., productivity or caregiver costs).

## Methods

### Analytical overview

With the R software package (version 2.13.0; R Development Core Team, Vienna, Austria), we used a previously developed Markov model to simulate the transition of a cohort of individuals with mRCC based on the clinical course [20]. We used this model to estimate and compare the lifetime direct medical costs and health outcomes associated with different first-line strategies for mRCC from the perspective of the Chinese healthcare system. The future costs and benefits were discounted using a 3% annual discount rate.

Although multiple agents for mRCC have been evaluated in clinical trials, the most commonly used first-line strategies are interferon-alfa, interleukin-2, sunitinib and bevacizumab [19]. Therefore, the cost and effectiveness of the five first-line strategies (sunitinib, bevacizumab plus interferon-alfa, interferon-alfa, interleukin-2 and interleukin-2 plus interferon-alfa) were evaluated and compared. Because of the absence of head-to-head clinical trials comparing these five first-line strategies, an indirect comparison was performed following a well-established approach [15], [17], [21]. Because the affordability of sunitinib in China can be a challenge, the Sunitinib Patient Assistance Program (SPAP) was introduced to make sunitinib available to eligible patients. Currently, the SPAP requires RCC patients to pay for three cycles of sunitinib, after which they will receive donations of sunitinib until the end of their treatment [22]. Therefore, the scenario analyses included the importance of SPAP for sunitinib.

Four types of parameters were inputs for the model: transition probabilities, which reflect the probabilities of moving between health states at each cycle; event proportions, which govern the ratios of events; direct medical costs, which were estimated based on health resource consumption; and health state utilities, which project the health-related quality of life for different health states. These data were derived from the published literature or from local health systems.

Cost-effectiveness ratios were calculated to evaluate the outcomes of the different strategies. The main health outcomes were presented by quality-adjusted life-year (QALY). The results were presented as an incremental cost-effectiveness ratio (ICER).

### Decision Model Structure

**Figure 1:**
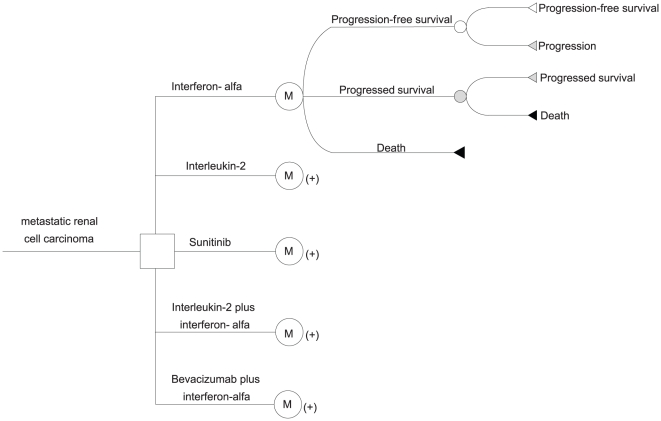
The model structure illustrating the five first-line strategies for treating mRCC.

A decision-analytic Markov model was used to compare the lifetime clinical progression, costs and utilities of treating mRCC with sunitinib, bevacizumab plus interferon-alfa, interferon-alfa, interleukin-2 and interleukin-2 plus interferon-alfa. Three discrete health states reflecting different characteristics of the disease were identified: progression-free survival (PFS), progressed survival (PS) and death. The structure of the model is shown in Figure 1. To be consistent with other economic evaluations in the literature, a 10-year time horizon was used to determine the lifetime outcomes [16], [17]. In the Markov model, the cycle length was 6 weeks and the entry state was progression-free survival. During each 6-week cycle, the patients either remained in their assigned health state or progressed to a new health state. The hypothetical patient demographics, when entering the model, matched those of the patients in the pivotal clinical trials: histologically proven renal cell carcinoma, advanced metastatic disease, finding of at least one measurable lesion, a World Health Organization (WHO) or Eastern Cooperative Oncology Group (ECOG) performance status (PS) of 0 or 1, and adequate organ function [5], [11], [12].

### Clinical data and adjusted indirect comparison

We performed a literature search in the following electronic databases for the pivotal clinical trials pertaining to mRCC treatments: PubMed, EMBASE, CINAHL, AltHealthWatch, the Cochrane Library, and the National Library of Science and Technology. The search covered the periods from the databases inceptions to the end of July 2011. However, no clinical trial directly comparing these five strategies was identified. Therefore, an indirect comparison of key clinical trials was performed. Clinical effectiveness data, including HR (hazard ratio), were separately extracted from the four multicenter, randomised-controlled clinical trials that had interferon-alfa as a common comparator; each of these trials constituted level 1 evidence [23].

The AVOREN Trial randomised 649 patients with mRCC to receive interferon-alfa 2a and bevacizumab (n = 327) or placebo and interferon-alfa 2a (n = 322). The median PFS was significantly longer in the bevacizumab plus interferon-alfa group than in the control group (10.2 vs. 5.4 months, HR 0.63, 95% CI 0.52–0.75, p = 0.0001). The median OS was also longer, although the difference was not statistically significant (23.3 vs. 21.3 months, HR 0.91, 95% CI 0.76–1.10, p = 0.336) [11], [14].

In the sunitinib trial, 750 mRCC patients were randomly assigned to sunitinib or to interferon-alfa. The median PFS in the sunitinib arm (11 months) was significantly longer than in the interferon-alfa arm (5 months), corresponding to an HR of 0.42 (95% CI 0.32–0.54, P<0.001). The median OS was greater in the sunitinib arm than in the interferon-alfa arm (26.4 vs. 21.8 months, HR 0.821, 95% CI 0.673–1.001, P  = 0.051) [12], [13].

The MRC RE04/EORTC GU 30012 trial enrolled 1,006 advanced mRCC patients who were randomly allocated (1 to 1) by minimisation to receive interferon-alfa alone or combination therapy with interferon-alfa, interleukin-2, and fluorouracil. After a median follow-up of nearly 37 months, the median OS was 18.8 months for the patients receiving interferon-alfa versus 18.6 months for those receiving the combination therapy (HR 1.05, 95% CI 0.90–1.21, p = 0.55). There was no evidence that the median PFS differed between the treatment groups (5.5 vs. 5.3 months, HR 1.02, 95% CI 0.89–1.16, p = 0.81) [5].

The clinical trial reported by Groupe Francais d'Immunotherapie enrolled 425 mRCC patients who were randomly assigned to receive interleukin-2, interferon-alfa alone or both. There was no significant difference in OS among the three groups. However, the PFS in the combined cytokine treatment group was significantly higher than in the other two groups (P = 0.01) [6]. The HR of the interleukin-2 group compared with the interferon-alfa group was derived from a previous economic study (HR of PFS 0.895 [95% CI 0.680–1.202], HR of OS 1.083 [95% CI 0.718–1.394]) [17].

**Figure 2:**
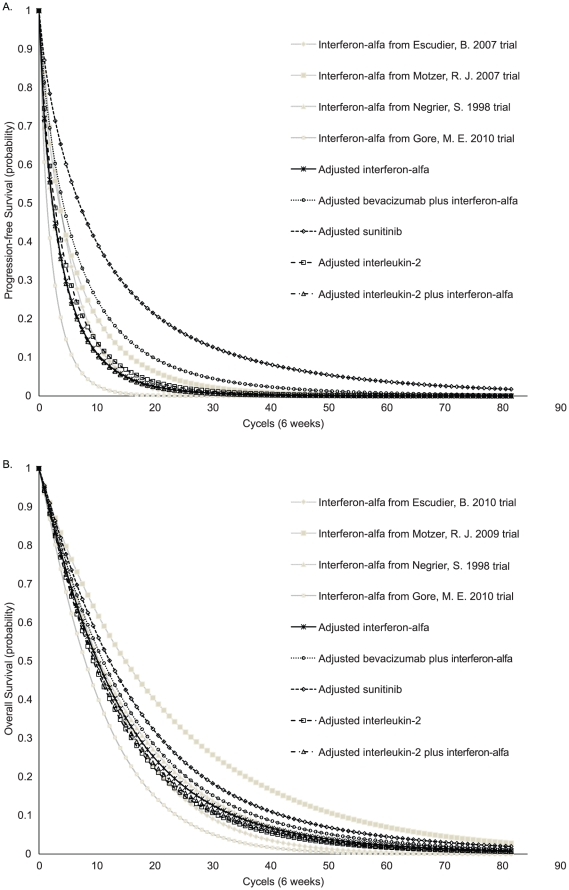
The Weibull plots of (A) progression-free survival and (B) overall survival.

Indirect comparisons of the five strategies were conducted using a hypothetical average interferon-alfa (reference) survival rate. Weibull survival models were fitted to the Kaplan-Meier PFS and OS data for interferon-alfa. The estimated Weibull parameters, scale (λ) and shape (γ), and their SEs and correlation coefficients are shown in Table 1. The variations in the interferon-alfa survival rates may be attributable to variations in baseline demographics or disease severities. Based on the Weibull model, the PFS and OS rates for interferon-alfa in the four trials were calculated at each cycle. The reference survival rates at each cycle were calculated and weighted according to patient numbers [21]. Once a survival rate was calculated, the survival rates for the active strategies were adjusted by the following formula: S_active strategies_ = (S_interferon-alfa(reference)_)^HR^. All HR data is presented in Table 2. We did not use the Weibull model to fit the Kaplan-Meier data of the other alternative strategies, because there are two important advantages to using the HRs to derive the survival curves, as mentioned by Hoyle, M. *et al* [16]. First, the method allows for the number of patients at risk on the Kaplan-Meier curve. Second, it allows for the analysis of uncertainty in clinical effectiveness by changing the HRs. The PFS and OS HRs between the alternative strategies and interferon-alfa were derived from the previously mentioned published studies. The final adjusted Weibull PFS and OS survival rates for the five strategies are shown in Figure 2.

**Table 1:** The parameters of the Weibull curves fitted to the interferon-alfa Kaplan-Meier survival data from four pivotal clinical trials.

	Scale		Shape				
Trials	Mean	SE	Mean	SE	Adjusted R^2^	Correlation Coefficient	Reference
Progression-free survival							
Negrier, S. 1998 trial	0.360096	0.0149185	0.7626	0.0237	0.9216	−0.99954	[6]
Escudier, B. 2007 trial	0.174161	0.004828	1.024	0.017	0.9862	−0.99962	[11]
Motzer, R. J. 2007 trial	0.2447073	0.0078566	0.7914	0.0215	0.9565	−0.99954	[12]
Gore, M. E. 2010 trial	0.2288074	0.0094682	0.8361	0.0215	0.9605	−0.99962	[5]
Overall survival							
Negrier, S. 1998 trial	0.064372	0.000922	0.9871	0.0066	0.9959	−0.99973	[6]
Motzer, R. J. 2009 trial	0.0476218	0.0012449	0.9666	0.0118	0.9928	−0.999673	[13]
Escudier, B. 2010 trial	0.043549	0.000682	1.015	0.007	0.9978	−0.99911	[14]
Gore, M. E. 2010 trial	0.038574	0.000943	1.099	0.011	0.9861	−0.99911	[5]

**Table 2:** HR and probabilities of SAEs.

			Treatmentstrategy			
Parameters	interferon-alfa	Bevacizumabplus interferon-alfa	Sunitinib	Interleukin-2	Interleukin-2 plus interferon-alfa	Source
HR						
PFS(95% CI)	-	0.63(0.52–0.75)	0.42(0.32–0.54)	0.895(0.68–1.202)	1.02(0.89–1.16)	[5], [6], [11], [12], [17]
OS(95% CI)	-	0.91(0.76–1.1)	0.821(0.673–1.001)	1.083(0.718–1.394)	1.05(0.9–1.21)	[5], [6], [13], [14], [17]
Probability of SAEs*						
Neutropenia(range#)	1(0.9–1.1)	4(3.6–4.4)	12(10.8–13.2)	5(4.5–5.5)	5(4.5–5.5)	[5], [6], [11], [12]
Anaemia(range#)	3(2.7–3.3)	3(2.7–3.3)	4(3.6–4.4)	2(1.8–2.2)	2(1.8–2.2)	[5], [6], [11], [12]
Thrombocytopenia(range#)	0.99(0.89–1.09)	2(1.8–2.2)	8(7.2–8.8)	0.99(0.89–1.09)$	0.99(0.89–1.09)$	[5], [6], [11], [12]
Nausea(range#)	2(1.8–2.2)	3(2.7–3.3)	4(3.6–4.4)	5(4.5–5.5)	5(4.5–5.5)	[5], [6], [11], [12]
Fatigue/Asthenia(range#)	18(16.2–19.8)	12(10.8–13.2)	7(6.3–7.7)	25(22.5–27.5)&	25(22.5–27.5)	[5], [6], [11], [12]
Hypertension(range#)	0.66(0.59–0.72)	3(2.7–3.3)	8(7.2–8.8)	4(3.6–4.4)	4(3.6–4.4)	[5], [6], [11], [12]
Proteinuria(range#)	0§	7(6.3–7.7)	0§	0§	0§	[5], [6], [11], [12]

* Probabilities are presented as percentages.

# The range is from 90% to 110% of the mean.

$ We assumed that the probabilities were similar to those in the AVOREN Trial.

& We assumed the that probabilities were similar to those in the Gore, M. E. 2010 trial.

§ Rare data were reported; we assumed that the probability of proteinuria was zero.

### Medical health resources and utilities

A Chinese healthcare system perspective was used to estimate the costs in the current study. Direct medical costs, such as first and second-line treatment-related medicines, radiological and laboratory examinations, management of serious adverse events (SAEs), physician visits, and BSC in the terminally ill, were included. Indirect costs (e.g., lost productivity or premature death) were not included [17]; the costs were converted into 2011 US dollars (Table 3). All the unit costs of the health resources were estimated using data from the local health system or the National Development and Reform Commission (NDRC) of China [24].

**Table 3:** Base-Case Cost Estimates ($, year 2011 values) and Utilities.

Parameter	Median Cost (US $)	Range* (US$)	Description and Reference
Cost			
Sunitinib per 12.5 mg	71.5	fixed	Local charge [24]
Bevacizumab per 100 mg	815.1	fixed	Local charge [24]
Interferon-alfa per 300 MU	6.5	4.2–6.9	Local charge [24]
Interleukin-2 per 50 MU	13.4	12–13.8	Local charge [24]
Sorafenib per 400 mg	64.5	fixed	Local charge [24]
Morphine sulphate per 300 mg	1.4	1.3–1.6	Local charge [24]
Drug administration	18.5	16.6–20.3	Local charge [24]
Routine follow-up of patients per unit	38.5	30.8–53.8	Local charge [24]
Expenditures of SAEs (per event)			
Neutropenia	461.5	415.4–507.7	Calculation
Anaemia	531.7	478.5–584.9	Calculation
Thrombocytopenia	3551.7	3196.5–3906.9	Calculation
Nausea	44.3	39.9–48.7	Calculation
Fatigue/Asthenia	115.4	103.8–126.9	Calculation
Hypertension	12.9	11.6–14.2	Calculation
Proteinuria	115.4	5.8–7.1	Calculation
Utilities			
Utility of PFS	0.65	0.26∼0.87	[16]
Utility of OS	0.47	0.19∼0.58	[16]

* The ranges of costs and utilities were obtained from local charge and literatures, respectively.

The drug costs associated with each strategy were estimated according to the following schedules. The interferon-alfa was assumed to be administered by subcutaneous injection three times per week in first cycle at 3 MU/dose in the first week, 6 MU/dose in the second week, and 9 MU/dose thereafter. The subsequent cycles involved three 9-MU/dose injections. The sunitinib was assumed to be administered orally at 50 mg once daily for 4 weeks, followed by 2 weeks off treatment. Bevacizumab 10 mg/kg or placebo was assumed to be administered intravenously every 2 weeks. Interleukin-2 was assumed to be administered intravenously as 18 MU×body-surface area (m^2^) daily for 5 days, once every 3 weeks. Sorafenib (a second-line treatment) was assumed to be administered orally at 400 mg twice per day. To estimate the dosage of the therapeutic agents, we assumed that a typical patient weighed 65 kg and had a height of 1.64 m, resulting in a body-surface area (BSA) of 1.72 m^2^. We assumed that the unused drugs in the open vials were discarded.

Based on a previous study [17], we assumed that 66% of patients would receive second-line treatment when disease progressed. Of those, nearly 33% received second-line targeted therapy (14.2%, sunitinib; 12.2% sorafenib; 1.7% bevacizumab; and 4.9% other targeted agents) [25]; 33.5% received either interferon-alfa or interleukin-2 [17], regardless of the first-line treatment. Because other targeted agents, such as everolimus and axitinib, are unavailable in the Chinese market, we assumed that the 4.9% receiving other targeted agents would switch to sorafenib, based on expert opinion. Because no detailed BSC treatment was reported in the clinical trial, we assumed that the major cost would be pain medications (morphine sulphate) and that the mean dosage would be 300 mg taken twice daily.

A previous study has shown that hematologic toxicities and fatigue/asthenia are the main drivers of management costs in sunitinib treatment and that proteinuria and fatigue/asthenia are the main drivers in bevacizumab plus interferon-alfa treatment [26]. Our model incorporated these treatment-related AEs. Other high incidence toxicities, such as nausea and hypertension, were also included. SAEs (grade≥3) management strategies were based on clinical practice and expert opinions. The incidences of SAEs were sourced from clinical trials (Table 2). The unit costs of treating SAEs were estimated based on patient records in local hospitals.

The utility values of the PFS and PS states were derived from previous published studies; 0.60 and 0.45 were assigned for PFS and PS, respectively. Their standard errors were estimated at 10% of the mean in our sensitivity analysis [16].

### Sensitivity Analyses

One-way deterministic sensitivity analyses were used to identify key model input parameters over the low/high values, which are listed and illustrated in Table 2 and Table 3. The results are presented as a tornado diagram based on the impact of the variable on the incremental net health benefit, using 3×the per capita GDP of China as the threshold according to World Health Organization(WHO) guidelines for cost-effectiveness analysis13,290 [27], [28], [29]. Probabilistic sensitivity analyses (PSA) were used to evaluate the impact of uncertainty across all the parameters simultaneously. The values of the input parameters were sampled from lognormal distributions for costs and from β distributions for utility values and probabilities or proportions with an assumed standard deviation of 10% from mean values. Using these distributions, 1,000 iterations of 1,000 simulated patients were run for our analysis. The results are shown on a cost-effectiveness plane. The outcomes projected from all 1,000 simulations were used to plot acceptability curves, which estimated the willingness to pay (WTP) threshold for an incremental unit of effectiveness.

## Results

### Base Case Result

The base case cost-effectiveness results (Table 4) were estimated with a 10-year time horizon. Much longer periods of PFS achieved by the targeted therapies (sunitinib and bevacizumab plus interferon-alfa) resulted in longer survival times with more QALYs than could be achieved with cytokine therapies (interferon-alfa, interleukin-2 and interleukin-2 plus interferon-alfa). The sunitinib strategy produced an average of 1.71 years in the PFS health state, compared to 0.59 years, 1.04 years, 0.67 years and 0.58 years for those receiving interferon-alfa, bevacizumab plus interferon-alfa, interleukin-2 and interleukin-2 plus interferon-alfa, respectively. The sunitinib strategy gained the greatest number of QALYs over the course of the disease (1.40), compared to 1.11 QALYs for the interferon-alfa strategy and 1.23 for the bevacizumab plus interferon-alfa strategy. The acquisition PFS time of sunitinib largely explains its higher QALY output.

**Table 4:** The base-case results for the five first-line therapies.

		Treatment strategy			
Model Outcome*	Interferon-alfa	Bevacizumab plus interferon-alfa	Sunitinib	Interleukin-2	Interleukin-2 plus interferon-alfa
Cost($) without SPAP					
in progression-free stage	6802.82	157562.65	81728.07	4184.96	10464.30
in progressed stage	25817.48	21302.31	14250.28	23256.96	25159.11
total	32620.30	178864.96	95978.35	27441.92	35623.41
Cost($) with SPAP					
in progression-free stage	6802.82	157562.65	15875.58	4184.96	10464.30
in progressed stage	19297.00	16075.92	11078.88	17484.91	18837.44
total	26099.82	173638.56	26954.46	21669.87	29301.74
Survival(Year)					
in progression-free stage	0.59	1.04	1.71	0.67	0.58
in progressed stage	1.82	1.51	1.00	1.64	1.77
total	2.41	2.55	2.71	2.32	2.35
QALYs					
in progression-free stage	0.35	0.61	0.98	0.40	0.34
in progressed stage	0.76	0.63	0.41	0.68	0.74
total	1.11	1.23	1.40	1.08	1.09
CER without SPAP	29285.33	145062.35	68765.81	25298.13	32798.34
CER with SPAP	23431.48	140823.65	19312.12	19977.00	26978.00
ICER without SPAP#	177724.92	1021196.49	220384.01		5872545.72
Comment without SPAP		Dominated			Dominated
ICER with SPAP#	152038.42	1024876.36	16992.99		5478038.63
Comment with SPAP	Extended dominated	Dominated			Dominated

Abbreviations: SPAP, patient assistance program; QALY, quality-adjusted life-year; ICER, incremental cost-effectiveness ratio; LY, life-year; dominated, another strategy was both more effective and less costly; extended dominated, another strategy achieved more effectiveness at a lower incremental cost-effectiveness ratio.

* All future costs and QALYs were discounted at 3%.

# Compared with Interleukin-2.

**Figure 3:**
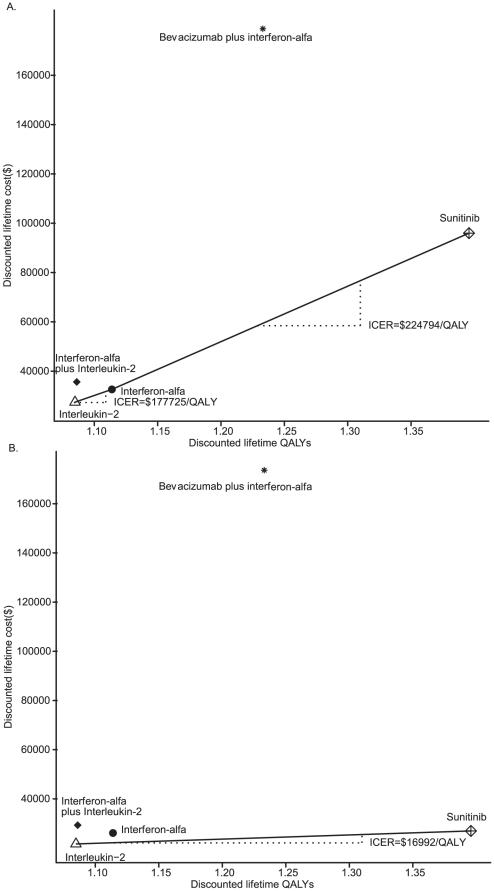
The cost-effectiveness of first-line strategies for mRCC patients. (A) without the SPAP; (B) with the SPAP. The x-axis indicates the discounted lifetime quality-adjusted life-years (QALYs) for each strategy, and the y-axis indicates the total discounted lifetime costs (in US dollars). The oblique line connects interleukin-2 and the most cost-effective strategies; strategies above the straight lines were dominated or extended dominated. In the cost-effective plane, the values of the most incremental cost-effectiveness ratios (ICER) are depicted.

Table 4 also presents the total direct costs incurred by each strategy. When no SPAP was offered, the targeted therapies were more expensive. The total cost of bevacizumab plus interferon-alfa was $178,864.96 ($157,562.65 for PFS), followed by $95,978.35 for sunitinib ($81,728.07 for PFS), $35,623.41 for interleukin-2 plus interferon-alfa ($10464.30), $32,620.30 for interferon-alfa ($6,802.82) and $27,441.92 for interleukin-2 ($4,184.96). The results of the scenario analysis indicated that the SPAP significantly reduced the cost of the progression-free stage for the sunitinib strategy ($15,875.58). The relative cost-effectiveness analyses showed that the bevacizumab plus interferon-alfa and interleukin-2 plus interferon-alfa strategies were both dominated because their incremental costs per QALY gained were $1,021,196.49 and $5,872,545.72, respectively (Table 4 and Figure 3A). Regarding the SPAP, the scenario analysis showed that the sunitinib strategy achieved dominance; the bevacizumab plus interferon-alfa and interleukin-2 plus interferon-alfa strategies were dominated, and the interferon-alfa strategy was extended dominated (Table 4 and Figure 3B).

### Sensitivity Analyses

**Figure 4:**
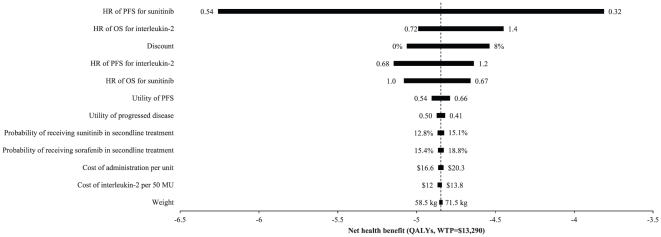
A tornado diagram representing the net health benefit (in QALYs, with WTP = $13,290). The diagram determined by a one-way sensitivity analysis of sunitinib vs. interleukin-2 for mRCC. The vertical line represents the base-case value for the net health benefit with WTP = $13,290. PFS: progression-free survival; OS: overall survival; HR: hazard ratio. The results from the one-way sensitivity analysis indicated that the most influential factor in the model was the 95% CI for the survival HR comparing sunitinib and interferon-alfa.

The tornado diagram (Figure 4) revealed that the net health benefit of sunitinib vs. that of interleukin-2 was sensitive to some of the input parameters; the PFS HR of sunitibinb was the most influential factor. Changing the PFS HR for sunitinib vs. interferon-alfa in the range of the 95% CI had the effect of changing the net health benefit significantly. At the upper boundary of the HR, which resulted in a shorter PFS with sunitinib, the net health benefit decreased to −6.26 QALYs (WTP = $13,290). A longer PFS for sunitinib was observed at the lower boundary of the HR, with the net health benefit increasing to −3.81 QALYs. The other important drivers of the model were the OS and PFS HR for interleukin-2, discount rate and utilities. Other factors, such as the costs of managing SAEs, had little impact.

**Figure 5:**
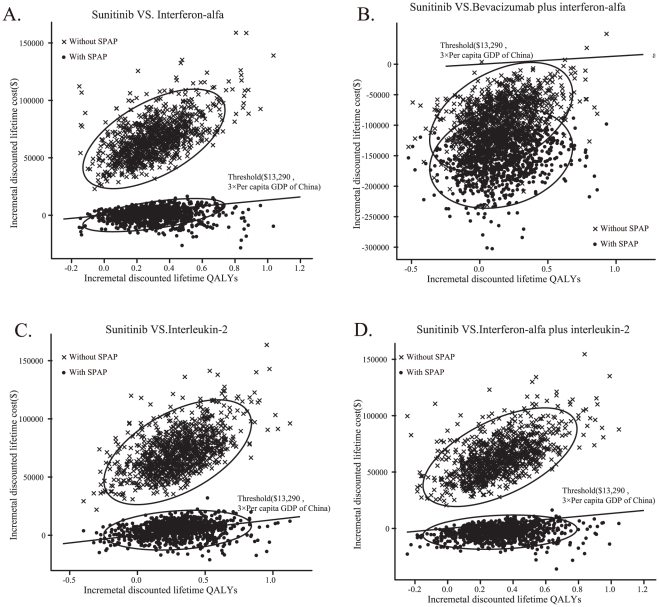
The probabilistic results of the incremental cost-effectiveness differences. The comparisons were conducted between sunitinib and (A) interferon-alfa, (B) bevacizumab plus interferon-alfa, (C) interleukin-2 and (D) interleukin-2 plus interferon-alfa for a cohort of 1,000 mRCC patients with or without the SPAP. The y-axis represents the incremental costs. The x-axis represents the incremental quality-adjusted life years (QALYs) gained. The ellipses surround 95% of the estimates. The dots found below the ICER threshold (the oblique lines) reflect simulations in which the cost per additional QALY gained with sunitinib was below the ICER threshold.

The plot data from the PSA of 1,000 simulations revealed the probabilities of meeting the ICER thresholds of $13,290 per additional QALY for sunitinib over bevacizumab plus interferon-alfa, interferon-alfa, interleukin-2 and interleukin-2 plus interferon-alfa. The results are shown in Figure 5. With the SPAP, the probabilities of achieving cost-effectiveness with sunitinib for interferon-alfa, bevacizumab plus interferon-alfa, interleukin-2 and interleukin-2 plus interferon-alfa were 75.2%, 100%, 45.1% and 95.3%, respectively, under the $13,290 threshold. Without the sunitinib patient assistant program, the probabilities of achieving cost-effectiveness with sunitinib were all zero when compared to all other options, except for bevacizumab plus interferon-alfa (∼99%).

**Figure 6:**
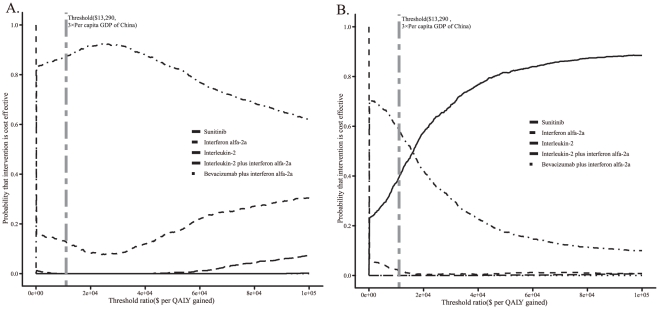
The cost-effectiveness acceptability curves for the five first-line strategies. (A) without the SPAP; (B) with the SPAP. The y-axis indicates the probability that a strategy is cost-effective across the willingness to pay per QALY gained (x-axis). The bold vertical dashed line represent the thresholds for China.

The cost-effectiveness acceptability curves (CEACs) showed the preferred first-line strategies for mRCC when accounting for a range of cost-per-QALY thresholds. The CEAC plot shows that when no patient assistant program was offered, interleukin-2 achieved a nearly 89% likelihood, when the threshold level was $13,290 (Figure 6A). When the program was offered, sunitinib achieved likelihoods of nearly 40% (Figure 6B). Sunitinib and interleukin-2 shared a likelihood of nearly 50% when the threshold was $16,000. In China, local governments have the power to add additional therapies ino basic medical services supplied by central government according to their economic development level [30], [31]. Although interleukin-2 achieved the majority of cost-effective probability at the threshold of 3×average per capita GDP of China, local governments are still expected to add new therapies. Table 5 listed the cost-effective probabilities of 5 alternative strategies for 32 Chinese provinces at 3×local per capita GDP.

**Table 5:** The cost-effective probabilities of five alternative strategies for 32 Chinese provinces with SPAP.*

Region	GDP($)	interferon-alfa	Bevacizumabplus interferon-alfa	Sunitinib	Interleukin-2	Interleukin-2plus interferon-alfa
Mainland China	4430	1.4	0	44.7	53.9	0
Shanghai	10828	0.5	0	71.2	28.3	0
Tianjin	10400	0.5	0	69.4	30.1	0
Beijing	10378	0.5	0	69.4	30.1	0
Jiangsu	7682	0.4	0	61.6	38	0
Zhejiang	7390	0.4	0	60.6	39	0
Inner Mongolia	6969	0.4	0	58.9	40.7	0
Guangdong	6440	0.5	0	56.7	42.8	0
Liaoning	6172	0.6	0	55	44.4	0
Shandong	6078	0.6	0	54.5	44.9	0
Fujian	5748	0.5	0	52.4	47.1	0
Jilin	4614	1.1	0	45.7	53.2	0
Hebei	4152	1.8	0	42.9	55.3	0
Hubei	4079	1.9	0	42	56.1	0
Chongqing	4043	1.9	0	41.8	56.3	0
Shaanxi	3966	1.8	0	41.5	56.7	0
Heilongjiang	3946	1.8	0	41.3	56.9	0
Ningxia	3853	1.9	0	40.7	57.4	0
Shanxi	3759	1.9	0	40.2	57.9	0
Xinjiang	3670	2	0	39.4	58.6	0
Henan	3605	2.1	0	39.1	58.8	0
Hunan	3576	2.1	0	39	58.9	0
Qinghai	3545	2.1	0	38.7	59.2	0
Hainan	3496	2.3	0	38.2	59.5	0
Jiangxi	3127	2.5	0	36.7	60.8	0
Sichuan	3104	2.5	0	36.6	60.9	0
Guangxi	3050	2.5	0	36.2	61.3	0
Anhui	3045	2.5	0	36.1	61.4	0
Tibet	2497	3.1	0	32.2	64.7	0
Gansu	2379	3.2	0	31.6	65.2	0
Yunnan	2320	3.4	0	31.2	65.4	0
Guizhou	1953	4.1	0	29.4	66.5	0

* The probabilities were estimated at threshold of 3×per capita GDP and presented as percentages, SPAP: sunitinib patient assistant program.

## Discussion

New targeted therapies for the first-line treatment of mRCC have increased survival rates and improved quality of life. Nevertheless, the widespread use of new targeted therapies comes with a dramatic increase in health care costs. A cost-effectiveness evaluation of the recommended first-line therapies in a health resource–limited setting is necessary to balance the economic burden with the health benefits. Using indirect comparison and decision-analytic modelling techniques, we estimated the cost-effectiveness of five first-line mRCC strategies over a lifetime horizon from the perspective of the Chinese healthcare system.

Our results suggest that targeted therapies as first-line treatment for mRCC provide more health benefits than do traditional cytokine therapies. Although the enhanced survival benefits resulting from the targeted therapies were not significant, the prolonged PFS survival improved the benefit of the targeted therapies over cytokines, which is an important reason why they are now covered by some developed countries [18]. Nevertheless, the gap between the costs of the targeted therapies and payment capacity in a health resource-limited setting is still large. The ICERs of sunitinib and bevacizumab plus interferon-alfa compared to cytokine therapies are far greater than the societal willingness-to-pay ($13,290for China). Interleukin-2 and interferon-alfa are still practical options in a resource-limited setting. An economic evaluation from the perspective of the UK NHS estimated that the cost per QALY ranged from $109,522 for sunitinib to $262,535 for bevacizumab plus interferon-alfa. The two therapies could not be considered cost-effective at a willingness-to-pay threshold of $46104/QALY, a result which is consistent with our findings [32]. Although sunitinib showed cost-effectiveness as a first-line mRCC treatment from the perspective of the US, with a threshold of $50,000 to $100,000 per LY or QALY, the results are not applicable to the developing regions of the world because their thresholds are far less than $50,000.

If the sunitinib patient assistance program were available to patients from poor regions, the sunitinib strategy might be the optimal alternative option when the threshold of the willingness-to-pay is greater than $16,000(Figure 6B). However, our analysis showed the threshold of sunitinib strategy with the program is still higher than the value of thrice the average Chinese GDP per QALY, which indicates that adding sunitinib into the basic medical service would not be cost-effective for Chinese central government. The Chinese mainland has 32 provinces, among which the per capita GDP differs significantly. In 2010, for example, the per capita GDP ranged from $1,953 in Guizhou province to $10,828 in the city of Shanghai as showing Table 5 [27]. Local government could consider covering sunitinib in their local supplemental medical service according to local economic development level. The results from Table 5 could supply the decision information for local governments.

At present, bevacizumab is also an alternative opion for mRCC in China, so the physicians, decision makers or patients would make the choice between sunitinib and bevacizumab. Our results showed sunitinib provides more health benefits for lower costs than bevacizumab plus interferon-alfa, regardless of the SPAP. Our findings are consistent with a recently published study based on the perspectives of the healthcare systems of the US and Sweden, which used indirect comparisons of survival data. The study showed that sunitinib as a first-line option for mRCC was a cost-effective alternative to bevacizumab plus interferon-alfa, because sunitinib was more effective and less costly [33].

The current study has some limitations. Using a Weibull distribution to extrapolate the outcomes beyond the time horizon of the trial follow-ups was an inevitable limitation of this analysis. The one-way sensitive analysis showed that the HR of the PFS and OS had important impacts on the final result. The short median follow-ups of the pivotal clinical trials did not provide enough survival data for a comparison with the median survival that was estimated by the model. At the same time, we did not consider the potential benefits of many of the uncertainties surrounding long-term survival rates, such as new therapeutic agents for second-line therapy, which might improve survival and life quality. Our results could be updated when long-term data are available. However, no randomised controlled trial has yet determined the long-term mRCC outcomes of first-line, second-line, or third-line therapies to the point of death. Therefore, it may be difficult to accurately measure the benefits of first-line therapy in future analyses, suggesting that a modelling technique may be the only realistic alternative.

Another potential limitation was the choice the Chinese healthcare system as our baseline perspective, which led to only direct medical costs being included in the model. Considering a societal perspective, which adds the additional burden of disease on families and caregivers and other indirect costs, may increase the costs associated with mRCC. As such, oral medications (e.g., sunitinib), prolonged PFS (e.g., sunitinib and bevacizumab) and reduced toxicity may produce more favourable results. There is no well-established method for incorporating such societal perspectives when measuring the impact of cost-effectiveness of first-line therapies for mRCC.

Because of the absence of head-to-head trials for the five first-line strategies for mRCC, an indirect comparison was used in the present analysis, which was another inevitable limitation. Similar cohort characteristics for the five strategies were assumed in our indirect comparison, and the results of the indirect comparison were imputed into the analytical model. Nevertheless, when no direct data is available, indirect comparisons using robust methods are accepted by many authors worldwide. Future research should directly compare the clinical efficacy of these strategies, especially those of different targeted therapies.

Other important limitations of the current economic analysis should be considered. In particular, we did not fully explore other therapeutic strategies for mRCC treatment, such as temsirolimus, everolimus and pazopanib, because they are still awaiting approval from the State Food and Drug Administration of China [10]. Targeted therapies have shown more favourable health benefits for certain subgroups [34]. Therefore, optimising the selection of the patients receiving targeted therapies could increase the cost-effectiveness of more expensive strategies. However, we did not present economic outcomes for such subgroups, because we were unable to adjust the five strategies in an indirect comparison applicable to such subgroup cohorts. Finally, utility values were obtained from literature published abroad and thus may not reflect Chinese data. However, opinions from Chinese urologists and oncologists suggest, quality of life of mRCC patients in China should not be significantly different from external mRCC patients. Although utilities have some impact on the result, the results of sensitivity analysis indicated that the influence is limited. Nevertheless, we are confident that the model faithfully represented the common clinical conditions of mRCC in a health resource–limited setting. We believe this study has the potential to be an important reference point for decision makers.

### Conclusion

In the Chinese healthcare setting, a representative health resource–limited region, traditional cytokine therapy is the cost-effective option. When the threshold is higher than $16,000, sunitinib might be a cost-effective therapy option compared to bevacizumab plus interferon-alfa, interferon-alfa, interleukin-2 and interleukin-2 plus interferon-alfa, based on its superior PFS benefit and Patient Assistance Program.

## References

[1] Rini BI, Campbell SC, Escudier B (2009). Renal cell carcinoma.. Lancet.

[2] Curti BD (2004). Renal cell carcinoma.. JAMA: the journal of the American Medical Association.

[3] Sengupta S, Zincke H (2005). Lessons learned in the surgical management of renal cell carcinoma.. Urology.

[4] Lam JS, Bergman J, Breda A, Schulam PG (2008). Importance of surgical margins in the management of renal cell carcinoma.. Nature clinical practice Urology.

[5] Gore ME, Griffin CL, Hancock B, Patel PM, Pyle L (2010). Interferon alfa-2a versus combination therapy with interferon alfa-2a, interleukin-2, and fluorouracil in patients with untreated metastatic renal cell carcinoma (MRC RE04/EORTC GU 30012): an open-label randomised trial.. Lancet.

[6] Negrier S, Escudier B, Lasset C, Douillard JY, Savary J (1998). Recombinant human interleukin-2, recombinant human interferon alfa-2a, or both in metastatic renal-cell carcinoma. Groupe Francais d'Immunotherapie.. The New England journal of medicine.

[7] Coppin C, Porzsolt F, Awa A, Kumpf J, Coldman A (2005). Immunotherapy for advanced renal cell cancer.. Cochrane database of systematic reviews.

[8] Gore ME, De Mulder P (2008). Establishing the role of cytokine therapy in advanced renal cell carcinoma.. BJU international.

[9] Hutson TE, Quinn DI (2005). Cytokine therapy: a standard of care for metastatic renal cell carcinoma?. Clinical genitourinary cancer.

[10] Sun M, Lughezzani G, Perrotte P, Karakiewicz PI (2010). Treatment of metastatic renal cell carcinoma.. Nature reviews Urology.

[11] Escudier B, Pluzanska A, Koralewski P, Ravaud A, Bracarda S (2007). Bevacizumab plus interferon alfa-2a for treatment of metastatic renal cell carcinoma: a randomised, double-blind phase III trial.. Lancet.

[12] Motzer RJ, Hutson TE, Tomczak P, Michaelson MD, Bukowski RM (2007). Sunitinib versus interferon alfa in metastatic renal-cell carcinoma.. The New England journal of medicine.

[13] Motzer RJ, Hutson TE, Tomczak P, Michaelson MD, Bukowski RM (2009). Overall survival and updated results for sunitinib compared with interferon alfa in patients with metastatic renal cell carcinoma.. Journal of clinical oncology : official journal of the American Society of Clinical Oncology.

[14] Escudier B, Bellmunt J, Negrier S, Bajetta E, Melichar B (2010). Phase III trial of bevacizumab plus interferon alfa-2a in patients with metastatic renal cell carcinoma (AVOREN): final analysis of overall survival.. Journal of clinical oncology : official journal of the American Society of Clinical Oncology.

[15] Ravasio R, Ortega C, Sabbatini R, Porta C (2011). Bevacizumab plus Interferon-alpha versus Sunitinib for First-Line Treatment of Renal Cell Carcinoma in Italy: A Cost-Minimization Analysis.. Clinical drug investigation.

[16] Hoyle M, Green C, Thompson-Coon J, Liu Z, Welch K (2010). Cost-effectiveness of temsirolimus for first line treatment of advanced renal cell carcinoma.. Value in health : the journal of the International Society for Pharmacoeconomics and Outcomes Research.

[17] Remak E, Charbonneau C, Negrier S, Kim ST, Motzer RJ (2008). Economic evaluation of sunitinib malate for the first-line treatment of metastatic renal cell carcinoma.. Journal of clinical oncology : official journal of the American Society of Clinical Oncology.

[18] Chabot I, Rocchi A (2010). How do cost-effectiveness analyses inform reimbursement decisions for oncology medicines in Canada? The example of sunitinib for first-line treatment of metastatic renal cell carcinoma.. Value in health : the journal of the International Society for Pharmacoeconomics and Outcomes Research.

[19] Molina AM, Motzer RJ (2011). Clinical practice guidelines for the treatment of metastatic renal cell carcinoma: today and tomorrow.. The oncologist.

[20] Wu B, Chen H, Shen J, Ye M (2011). Cost-effectiveness of adding rh-endostatin to first-line chemotherapy in patients with advanced non-small-cell lung cancer in china.. Clinical therapeutics.

[21] Kielhorn A, Porter D, Diamantopoulos A, Lewis G (2008). UK cost-utility analysis of rituximab in patients with rheumatoid arthritis that failed to respond adequately to a biologic disease-modifying antirheumatic drug.. Current medical research and opinion.

[22] Sutent® Patient Assistance Program.. http://www.chinacancernet.org.cn/Info/SortContentEn.asp?ID=31.

[23] Barbui C, Dua T, van Ommeren M, Yasamy MT, Fleischmann A (2010). Challenges in Developing Evidence-Based Recommendations Using the GRADE Approach: The Case of Mental, Neurological, and Substance Use Disorders.. Plos Medicine.

[24] National Development and Reform Commission (NDRC) .. http://en.ndrc.gov.cn/.

[25] Vickers MM, Choueiri TK, Rogers M, Percy A, Finch D (2010). Clinical outcome in metastatic renal cell carcinoma patients after failure of initial vascular endothelial growth factor-targeted therapy.. Urology.

[26] Mickisch G, Gore M, Escudier B, Procopio G, Walzer S (2010). Costs of managing adverse events in the treatment of first-line metastatic renal cell carcinoma: bevacizumab in combination with interferon-alpha2a compared with sunitinib.. British journal of cancer.

[27] List of Chinese administrative divisions by GDP per capita.. http://en.wikipedia.org/wiki/List_of_Chinese_administrative_divisions_by_GDP_per_capita.

[28] Eichler HG, Kong SX, Gerth WC, Mavros P, Jonsson B (2004). Use of cost-effectiveness analysis in health-care resource allocation decision-making: how are cost-effectiveness thresholds expected to emerge?. Value in health : the journal of the International Society for Pharmacoeconomics and Outcomes Research.

[29] Murray CJ, Evans DB, Acharya A, Baltussen RM (2000). Development of WHO guidelines on generalized cost-effectiveness analysis.. Health economics.

[30] Hougaard JL, Osterdal LP, Yu Y (2011). The Chinese healthcare system: structure, problems and challenges.. Applied health economics and health policy.

[31] Wagstaff A, Yip W, Lindelow M, Hsiao WC (2009). China's health system and its reform: a review of recent studies.. Health economics.

[32] Thompson Coon J, Hoyle M, Green C, Liu Z, Welch K (2010). Bevacizumab, sorafenib tosylate, sunitinib and temsirolimus for renal cell carcinoma: a systematic review and economic evaluation.. Health technology assessment.

[33] Benedict A, Figlin RA, Sandstrom P, Harmenberg U, Ullen A (2011). Economic evaluation of new targeted therapies for the first-line treatment of patients with metastatic renal cell carcinoma.. BJU international.

[34] de Reijke TM, Bellmunt J, van Poppel H, Marreaud S, Aapro M (2009). EORTC-GU group expert opinion on metastatic renal cell cancer.. European journal of cancer.

